# Temporary Hospitals

**Published:** 1892-05-28

**Authors:** 


### The Institutional Workshop.

### HOSPITAL CONSTRUCTION.

### TEMPORARY HOSPITALS.

One of the facts most clearly established by Dr. Thorne Thorne, in his report on the use and influence of hospitals for infectious diseases, is that buildings of a temporary nature, or which have been hurriedly put up under the influence of panic, have proved invariably to be unsatisfactory or inefficient in use. This is perhaps more specially true of iron buildings than of any others. The extreme difficulty of preserving an equable temperature, and of properly ventilating a building whose walls and roof are made of thin sheet-iron, is very forcibly impressed upon those who are in the habit of attending temporary churches built in this way. The ease with which an iron building can be put up, and its cheapness, are very potent inducements to a local authority to effect an apparent saving of public money at the expense of the permanent usefulness of the hospital. It is not surprising, therefore, to find an enterprising firm of builders of iron houses putting forth plans of hospitals of varying sizes, which they are prepared to supply on the very shortest notice. When, however, we find it stated that their plans are approved by the Local Government Board, it is time to utter a word of protest. In the first place, it is perfectly clear from the official publications of the Board that they strongly deprecate the use of iron for hospitals at all. It is, as we have said, extremely difficult to warm and ventilate properly a ward formed of iron. Further, iron buildings can only be regarded as of a temporary nature, and experience has proved over and over again that temporary buildings for the isolation of infectious diseases are extremely unsatisfactory and inadequate. Reverting again to the particular plans we referred to, we find the floor space per bed is wholly insufficient. In nearly every case the floor space is at the rate of 100 feet per bed, the recognised amount being 144 feet, and the dimensions recommended officially by the Local Government Board giving 156 feet. The provision for administrative purposes is ludicrously inadequate. In the most complete plan given, the total sleeping accommodation for staff consists of a room for a Matron! Apart, however, from the mere question of planning, which is, of course, susceptible of improvement, it is certain that in no case where permanent provision for infectious disease ought to be made is an iron building suitable for the purpose; and if, in view of a sudden epidemic, it becomes necessary to provide, within a short space of time, such temporary accommodation as can be quickly put up, iron buildings are resorted to, it should be on the distinct understanding that they were to be replaced by proper permanent buildings when the necessity for their use had ceased. But this is only to express again, in altered terms, the oft-repeated truth that it is the duty of all authorities, who have in any way to deal with outbreaks of infectious fevers, to provide adequate permanent accommodation without waiting for an epidemic to stir them into fitful panic-stricken action.

